# POU4F3 Acts as a Tumor Suppressor in Lung Adenocarcinoma via the Endoplasmic Reticulum Stress Signaling Pathway

**DOI:** 10.7150/jca.61660

**Published:** 2022-01-01

**Authors:** Xiaoxue Chai, Xuchao Ding, Xinran Lyu, Hongtao Zhao, Peiluo Huang, Juan Du, Lili Cao

**Affiliations:** 1 Oncology Department, Shandong Qianfoshan Hospital, Cheeloo College of Medicine, Shandong University, Jinan 250014, China.; 2 Shandong Provincial Key Laboratory for Rheumatic Disease and Translational medicine, the First Affiliated Hospital of Shandong First Medical University & Shandong Provincial Qianfoshan Hospital, Jinan 250014, China.; 3 Department of Immunology, Guilin Medical University, Guilin 541004, Guangxi, China.

**Keywords:** LUAD, POU4F3, ERS, Proliferation, Apoptosis

## Abstract

Lung cancer is the most common malignancy worldwide, and LUAD is the primary type of lung cancer. Recently, the POU transcription factor family has been associated with the development of multicancer, especially lung cancer. However, the relationship between POU domain class Ⅳ transcription factor Ⅲ (POU4F3) and lung cancer remains unknown. We detected the expression of POU4F3 in human LUAD and adjacent tissues with immunohistochemical staining, and we found that POU4F3 was expressed less in LUAD tissues compared with adjacent tissues. Patients with higher POU4F3 expression have more prolonged overall survival. We then constructed SPCA1 and A549 cells with stable overexpression or inhibition of POU4F3. We found that overexpressed POU4F3 suppressed LUAD cell proliferation *in vitro* and *in vivo*, according to CCK-8, colony formation, and xenograft assays. LUAD cell apoptosis was suppressed by POU4F3 overexpression based on Flow cytometry. The downregulation of POU4F3 yielded the opposite patterns. Next, we explored the possible mechanisms through which POU4F3 promoted the apoptosis of LUAD cells. Western blotting suggested that overexpression of POU4F3 significantly increased protein expression levels of the PERK/eIF2α/ATF4/CHOP and IRE1α/XBP1s pathways of ERS, while POU4F3 absence reversed the expressions of the above essential proteins in ERS pathways in SPCA1 and A549 cells. However, we found that PERK inhibitor but not IRE1 inhibitor can reverse the effect of POU4F3 overexpression on apoptosis. This study indicated that POU4F3 may work as a tumor suppressor in LUAD via regulating the PERK/eIF2α/ATF4/CHOP pathway. We made it possible to develop POU4F3 as a diagnostic, therapeutic, and prognostic target of LUAD.

## Introduction

Lung cancer is the most common malignancy worldwide, and lung adenocarcinoma (LUAD) comprises around 85% of all lung cancer 1. LUAD has a poor prognosis with a 5-year survival rate of less than 15 percent 2. The molecular mechanism for the occurrence and development of LUAD has not been illustrated in detail. Molecular targeted therapy for lung cancer has demonstrated remarkable clinical success in recent years. Therefore, identifying efficient biomarkers can facilitate the diagnosis and prognostic assessment of LUAD.

POU4F3, also known as Brain-specific homeobox protein 3C (Brn-3C), is a transcription factor with a highly conserved DNA binding domain 3. POU4F3 is located on human chromosome 5q31-q33 4, and its encoded protein expresses in the nucleus 5. POU4F3 is not only linked to hearing loss 6-8 and glaucoma 9 but also tumorigenesis. Leonard JH et al. reported that the absence of POU4F3 in Merkel cell carcinoma may indicate an aggressive disease 10. Promoter sequence methylation of the POU4F3 gene is also significantly increased in Cervical Intraepithelial Neoplasiacases 11, 12 and astrocytoma 13. POU4F3 is closely related to the occurrence of various tumors, but the biological role and clinical value of POU4F3 in LUAD remain unclear.

The purpose of this study was to investigate the relationship between POU4F3 and LUAD and possible molecular mechanisms. We focused on the effects of POU4F3 on the proliferation and apoptosis of LUAD to search for potential therapeutic targets for LUAD.

## Materials and methods

### Tissue samples

Tissue microarray slides included 92 LUAD tissue samples and 88 para-carcinoma tissue samples with clinicopathologic information and were purchased from Outdo Biotech (Shanghai, China) under the approval of the Ethics Committee of Shandong Qianfoshan Hospital (approval no. 2018-S0085). All patients provided written informed consent for clinical treatment under the regulations of the Declaration of Helsinki. According to the seventh edition of the American Joint Committee on Cancer (AJCC) system for lung cancer, all patients were clinically staged.

### Immunochemical staining (IHC)

The tissue slices were dewaxed, hydrated, and retrieved with 0.1% sodium citrate buffer. Following peroxidase blocking with 3% hydrogen peroxide, slices were incubated with rabbit anti-POU4F3 antibody (1:50, AV33065, Sigma) overnight at 4℃. The pieces were then incubated with IgG-HRP (mouse/rabbit) (#PV-9000, OriGene, China) at room temperature for 1 h. Three 3-diaminobenzidine tetrahydrochloride and hematoxylin were used to develop and counterstain the nuclei, respectively. The Zeiss microscope (Germany) was utilized to take images. The intensity score was obtained under a 50-fold field of vision (negative: 0; weak positive: 1; medium positive: 2; strong positive: 3). Three images in each section under a 400-fold field of vision were taken randomly to determine the staining percentage of positive cells (less than 5%: 0; 5%-25%: 1; 26%-50%: 2 points; greater than 51%: 3). Finally, the two fractions were multiplied to obtain the IHC score, which in turn can be used to evaluate the expression of POU4F3. Samples were divided into POU4F3-positive (≥4) and POU4F3-negative (<4) groups according to the median IHC score of LUAD and normal tissues.

### Cells and lentivirus transfection

Human LUAD cell lines SPCA1 and A549 were purchased from the Institute of Biochemistry and Cell Biology of the Chinese Academy of Sciences (Shanghai, China). All cell lines were cultured with DMEM (Gibco, USA), including 10% Fetal Bovine Serum (FBS, Gibco, USA) and 1% penicillin/streptomycin mix (Gibco, USA) under strict sterilization conditions at 37℃ with 5% CO_2_. The SPCA1 and A549 cell lines were authenticated using Short Tandem Repeat (STR) analysis, and regular mycoplasma testing was performed to prevent contamination. POU4F3 overexpression lentivirus (Lv-POU4F3) and its negative control lentivirus (Lv-control) were produced by Genomeditech (Shanghai, China). POU4F3 knockdown lentivirus (shPOU4F3) and its control lentivirus (control shRNA) were constructed by GeneChem (Shanghai, China). SPCA1 and A549 cells transfected with the above lentivirus were filtrated with puromycin (1.5 μg/mL) to obtain stable cell clones. The transfection efficiency was verified by quantitative real-time PCR (qRT-PCR) and Western blotting (WB).

### Quantitative PCR (qPCR)

Total RNA was extracted from stably transfected cells with a Trizol reagent (Invitrogen, USA). Total RNA (1 μg) was reverse-transcribed into first-strand complementary DNA using a PrimeScript® RT Reagent Kit (TaKaRa, Japan). PCR was performed with a SYBR Green PCR Kit (TaKaRa, Japan) using Bio-Rad iQ5. Primers for POU4F3 were listed as follows: POU4F3 Forward: 5'-AGTCTCTCACTCTCTCGCACAA-3', POU4F3 Reverse: 5'- GCTGTTCTTCTCTCGGTAGGC-3'; β-actin Forward: 5'-AGTTGCGTTACACCCTTTC-3', β-actin Reverse: 5'-CCTTCACCGTTCCAGTTT-3'. All samples were performed in triplicate and normalized to β-actin levels. Gene-specific relative mRNA levels were calculated by the standard equation 2^-(△CT sample-△CT control)^.

### CCK-8 assay

5×10^3^ cells were seeded in 96-well plates to assess proliferation rates. Each group had six replications. A Cell Counting Kit-8 (#CK04, Dojindo, Japan) was applied to cells for 1.0 h on the 0th, 24th, 48th, and 72nd h at 37℃. Next, the Optical Density (OD) was measured at 450 nm with a microplate reader. The immediate proliferation rate = (immediate OD - initial OD)/ initial OD.

### Colony formation assays

One thousand cells were seeded in 6-well plates and inoculated for ten days. The colonies were then fixed with methanol for 20 min and stained with crystal violet at room temperature for 20 min. ImageJ was used to count the cell clones.

### Flow cytometry

Cells were treated with 10μg/mL of tunicamycin (#T8480, Solarbio, China) and then treated with 1.0 μM GSK2606414 (the PERK inhibitor, Selleck Chemicals, USA), 50 μM STF083010 (the IRE1 inhibitor, MedChemExpress, USA), 20 μM Z-VAD-FMK (apoptotic inhibitor, MedChemExpress, USA), or DMSO for 48 h. Tunicamycin, a natural antibiotic, triggers cancer cell apoptosis 14 and has been diffusely applied to the study of tumor cell apoptosis and programmed cell death. All cells were treated with Annexin V-APC and 7-AAD (MultiSciences, China) at room temperature for 30 mins. The percentage of apoptotic LUAD cells was detected by flow cytometry (Becton Dickinson, USA).

### Xenograft assay *in vivo*

Male 4-week-old BALB/c (nu/nu) nude mice were purchased from Vital River Lab Animal Technology Co., Ltd. (Beijing, China). The animal research was approved by the Ethics Committee of Shandong Qianfoshan Hospital (approval no. 2018-S0085). 8×10^7^ cells suspended in 100 μL PBS were injected subcutaneously into the mice (n = 6). Thirty-five days after injection, the nude mice were euthanized under anesthesia. Tumors were surgically excised and weighted.

### Western blotting

SPCA1 and A549 cells with stable POU4F3 overexpression or knockdown were treated with ERS inducer tunicamycin for 48 h. The total cell protein was exacted with a modified RIPA buffer (Beyotime, China) on ice for 30 min and quantified with the BCA kit box (Beyotime, China). Equal amounts of proteins were separated by 10% SDS-PAGE (Epizyme, Shanghai) and transferred onto PVDF membranes (Millipore, America). After being blocked with 5% bovine serum albumin, the membranes were incubated with the primary antibodies POU4F3 (1:500, AV33065, Sigma-Aldrich, Germany), PERK (1:1000, 5683P, Cell Signaling Technology (CST), USA), p-PERK (1:1000, 3179S, CST, USA), EIF2α (1:1000, 9722S, CST, USA), p-EIF2α (1:1000, 9721S, CST, USA), ATF4 (1:500, AF5416, Affinity, China), CHOP (1:1000, 2895S, CST, USA), IRE1-α (1:1000, 3294T, CST, USA), p-IRE1-α (1:1000, AF7150, Affinity, China), XBP1s (1:1000, 83418S, CST, USA), Caspase3 (1:1000, 9662S, CST, USA), Cleaved caspase3 (1:1000, 9662S, CST, USA), and β-tubulin (1:1000, 2146S, CST, USA) at 4℃ overnight. Membranes were then incubated with suitable secondary antibodies goat anti‑mouse IgG (1:3000, 7076S, CST, USA) or goat anti‑rabbit IgG (1:3000, 7074S, CST, USA) at room temperature for one h. ECL chemiluminescence (Millipore, America) was utilized to detect the blots. Protein levels were normalized to β-tubulin. ImageJ was applied to measure the gray value of the strip. Relative protein expression = (the experimental group's target protein/the experimental group's β-tubulin)/ (the control group's target protein/the control group's β-tubulin).

### Statistical analysis

All experiments were conducted three times and analyzed with GraphPad Prism 8. The results were expressed as Mean ± SEM. The t-test was used for comparison between two groups. One-way ANOVA was used for comparison in more than two groups. Chi-square analyzed the positive rate of POU4F3 expression as well as the relationship between POU4F3 expression and clinicopathologic characteristics. Survival analysis was carried out through the Kaplan-Meier with the Log-rank (Mantel-Cox) test. *P* < 0.05 was considered statistically significant in all cases.

## Results

### POU4F3 expression is downregulated in LUAD tissues

**Figure 1:**
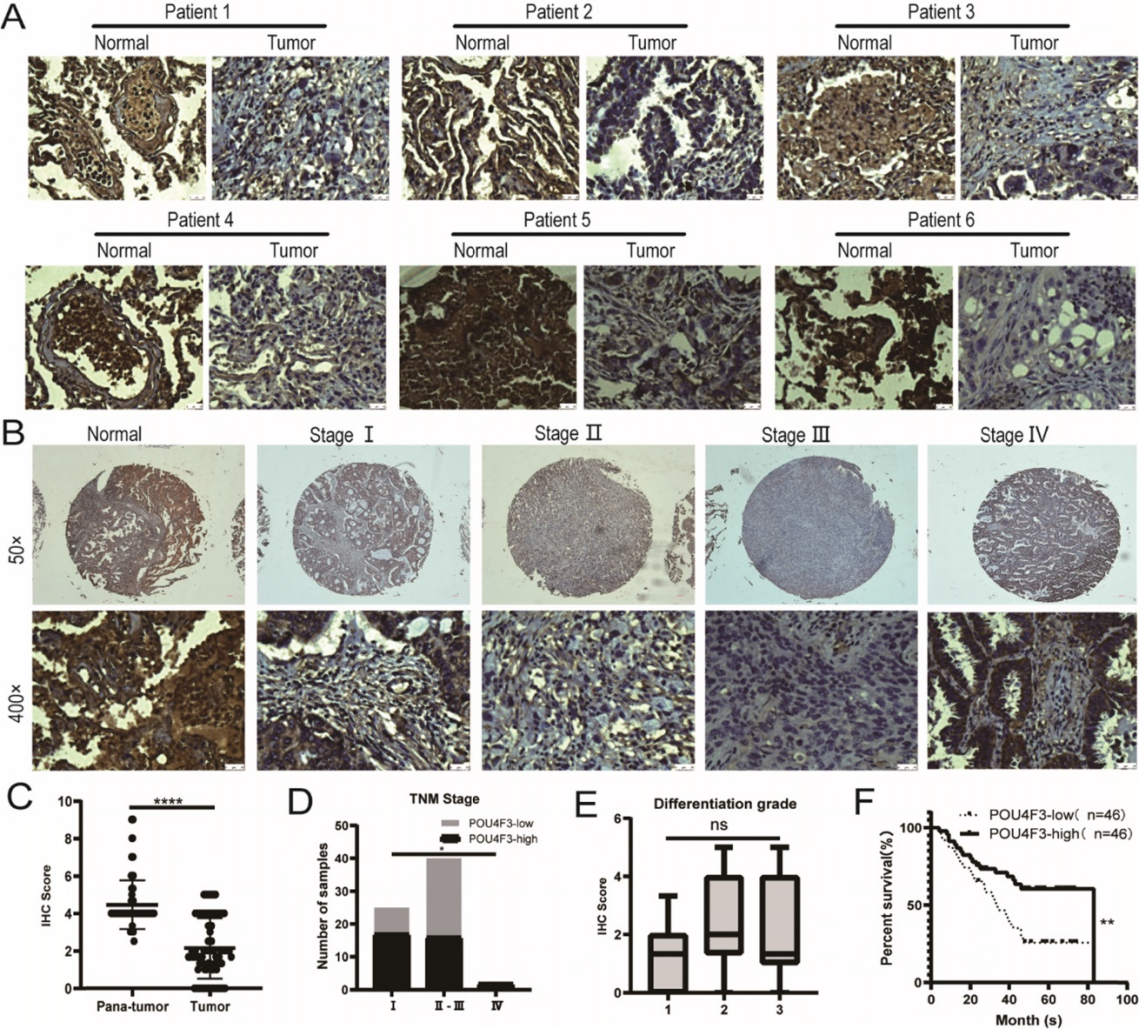
POU4F3 is low-expressed in LUAD and associated with clinicopathologic characteristics of LUAD. A/C: IHC indicates that POU4F3 expression was lower in LUAD tissues than in normal lung tissues (*P* < 0.0001). POU4F3 was distributed in both the nucleus and cytoplasm. B/D. The expression of POU4F3 in LUAD decreases with the increase of the TNM stage (*P* < 0.05). E. POU4F3 has no significant effect on the pathological grade of LUAD. F. Kaplan-Meier curves with Log-rank (Mantel-Cox) test for LUAD patients with low POU4F3-expressing (n= 46) versus high POU4F3-expressing tumors (*n*= 46) (*P*=0.0053). '****', '***', '**' and '*' represent *P* < 0.0001, 0.001, 0.01 and 0.05 respectively.

The expression of POU4F3 in 92 LUAD tissues and 88 normal lung tissues was detected with IHC, indicating that the degree of POU4F3 in LUAD tissues was lower than in normal tissues (Fig. 1A; *P* < 0.0001, Fig. 1C). POU4F3 is widely distributed in both the nucleus and cytoplasm. The positive rate of POU4F3 in LUAD tissues (26.09%) and in adjacent normal tissues (92.05%) was statistically significant (*P* < 0.0001, Table 1).

### POU4F3 expression is related to clinicopathological features and prognosis

The primary clinical characteristics of the 92 patients are shown in Table 2. Considering the overall low expression of POU4F3 in cancer tissues, we divided LUAD patients into the POU4F3-high (≥2) and POU4F3-low (<2) groups according to the median IHC score of POU4F3 expression in LUAD tissues, and we did so in order to evaluate the relationship between POU4F3 expression and clinicopathological characteristics. In terms of the TNM stage, Chi-square with Yates' correction showed that the differences in the TNM stage between the POU4F3-high and POU4F3-low groups were statistically significant (*P* = 0.0384, Table 2; *P* < 0.05, Fig. 1D). Tapering expression levels of POU4F3 could be observed from the TNM stage-I to stage-III (Fig. 1B). The Chi-square test showed a statistically significant difference in the POU4F3 positive rate between death and patients alive at the termination of the experiment (*P* = 0.0031, Table 2). Kaplan-Meier indicated that higher POU4F3 was associated with more prolonged overall survival (*P* < 0.0001, Fig. 1F). The median overall survival time was 40.5 months among 92 LUAD patients. However, the differences in age, gender, tumor size, tumor location, differentiation grade (*P* > 0.05, Fig. 1E), or lymph node metastasis between the POU4F3-high and the POU4F3-low groups were not statistically significant (*P* > 0.05, Table 2).

### POU4F3 decreases the proliferation of LUAD cells *in vitro*

**Figure 2:**
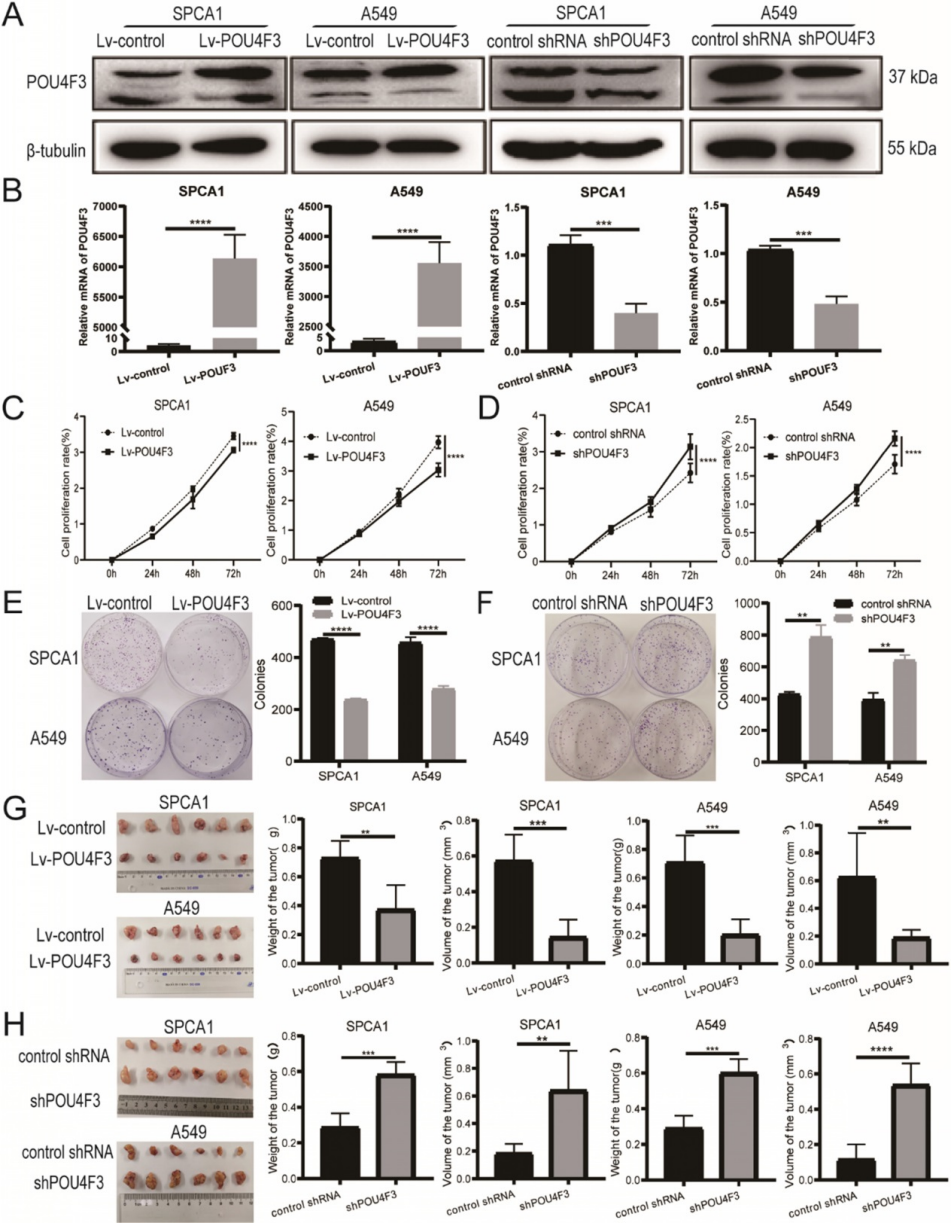
POU4F3 inhibited LUAD cell proliferation *in vitro* and* in vivo*. A/B. The transfection efficiency of POU4F3 overexpressed lentivirus or POU4F3 knockdown lentivirus in SPCA1 and A549 cells were detected by Western blotting (A) and qPCR (B). CCK-8 (C) and cell clone formation assays (E) illustrated that overexpressed POU4F3 significantly suppressed the proliferation of SPCA1 and A549 cells (*P* < 0.0001). CCK-8 (D) and cell clone formation assays (F) illustrated that POU4F3 knockdown significantly increased the proliferation of SPCA1 and A549 cells (*P* < 0.01). G. Xenograft assay showed volume and weight of the subcutaneous tumor in the overexpressed POU4F3 group were lower than that in the control group (*P* < 0.05). H. Xenograft assay showed volume and weight of the subcutaneous tumor in the POU4F3 knockdown group was higher than that in the control group (*P* < 0.05). '****', '***', '**' and '*' represent *P* < 0.0001, 0.001, 0.01 and 0.05, respectively.

To further investigate the biological function of POU4F3 in LUAD, we established stably overexpressed or inhibited POU4F3 cell models in SPCA1 and A549 cells, and we verified their transfection efficiency by WB (Fig. 2A) and qPCR (Fig. 2B). CCK-8 assays showed that compared with the control groups, POU4F3 overexpression significantly inhibited the proliferation rate in SPCA1 (*P* < 0.0001, Fig. 2C left) and A549 cells (*P* < 0.0001, Fig. 2C right). Also, the colony formation assays demonstrated that elevated POU4F3 decreased the colony formation numbers of LUAD cells (*P* < 0.0001, Fig. 2E). Additionally, POU4F3 knockdown significantly facilitated the LUAD cell proliferation according to CCK-8 (*P* < 0.0001, Fig. 2D) and colony formation assays (*P* < 0.01, Fig. 2F). In sum, CCK-8 and colony formation assays revealed that POU4F3 inhibited the proliferation of LUAD cells *in vitro*.

### POU4F3 restrains the growth of LUAD xenografts *in vivo*

SPCA1 and A549 cells with stably overexpressed POU4F3 or a control lentivirus were implanted subcutaneously into nude mice. The xenograft tumors weighed less and were smaller in the Lv-POU4F3 group than negative controls (*P* < 0.01, Fig. 2G). Likewise, tumors with stable POU4F3 knockdown illustrated that POU4F3 inhibition potentiated tumor growth (P < 0.001, Fig. 2H). All of the nude mice survived until the end of the experiment.

### POU4F3 facilitates tunicamycin-induced cell apoptosis

**Figure 3:**
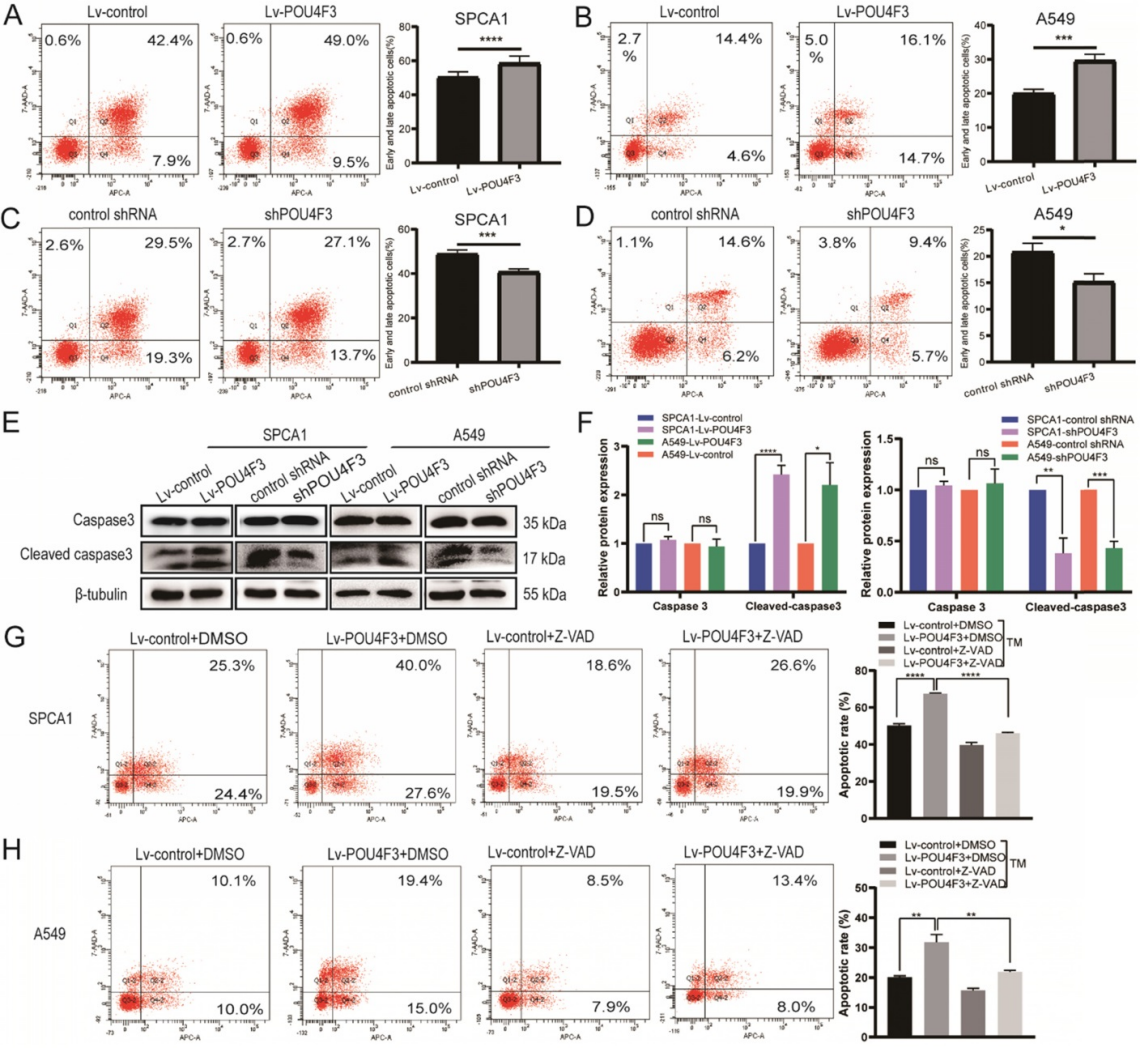
POU4F3 facilitated LUAD cell apoptosis. A/B. The early and late apoptotic proportion of SPCA1 (*P* < 0.0001) and A549 (*P* < 0.001) cells with or without POU4F3 overexpression. C/D. Under the same experimental conditions, the overall apoptosis proportion of SPCA1 (*P* < 0.001) and A549 (*P* < 0.05) cells was displayed with or without POU4F3 knockdown. E/F. WB was used to detect apoptosis-related protein Caspase3 and Cleaved caspase3. G/H. Flow cytometry was used to detect the apoptotic rate in SPCA1 (G) and A549 (H) with or without the apoptosis inhibitor Z-VAD. Z-VAD, a cell-permeable caspase inhibitor. '****', '***', '**', and '*' represent *P* < 0.0001, 0.001, 0.01, and 0.05 respectively.

To determine whether POU4F3 contributed to the apoptosis of LUAD, we analyzed the apoptosis of SPCA1 and A549 cells with stable POU4F3 overexpression or knockdown. Flow cytometry results showed that POU4F3 overexpression potentiated the total apoptosis percentage of SPCA1 (*P* < 0.0001, Fig. 3A) and A549 cells (*P* < 0.001, Fig. 3B). Likewise, POU4F3 knockdown yielded the reverse phenotype in SPCA1 (*P* < 0.001, Fig. 3C) and A549 cells (*P* < 0.05, Fig. 3 D). WB was also applied to examine the expression of apoptosis-related indicators Caspase3 and Cleaved caspase3 (Fig. 3E). We found that Cleaved caspase3 was promoted by POU4F3 overexpression and was weakened by POU4F3 inhibition in SPCA1 (*P* < 0.0001, Fig. 3F left) and in A549 (*P* < 0.0001, Fig. 3F right). However, the total Caspase3 was not changed by POU4F3 overexpression or inhibition (*P* > 0.05, Fig. 3F). Changes in Cleaved caspase3 expression levels further confirmed that POU4F3 promoted LUAD cell apoptosis.

To extend our investigation on the effects of POU4F3 on apoptosis in LUAD cells, the apoptosis inhibitor Z-VAD was used to interrupt the apoptotic pathway activated by POU4F3 overexpression. Flow cytometry results showed that the total apoptotic percentage in the Lv-POU4F3+DMSO group is higher than that in the Lv-control+DMSO group in SPCA1 (*P* < 0.0001, Fig. 3G) and A549 cells (*P* < 0.01, Fig. 3H). Meanwhile, the apoptotic rate induced by POU4F3 overexpression decreased after Z-VAD treatment in both of the LUAD cell groups (*P* < 0.01, Fig. 3G, H).

### POU4F3 may inhibit LUAD via the ERS pathways

**Figure 4:**
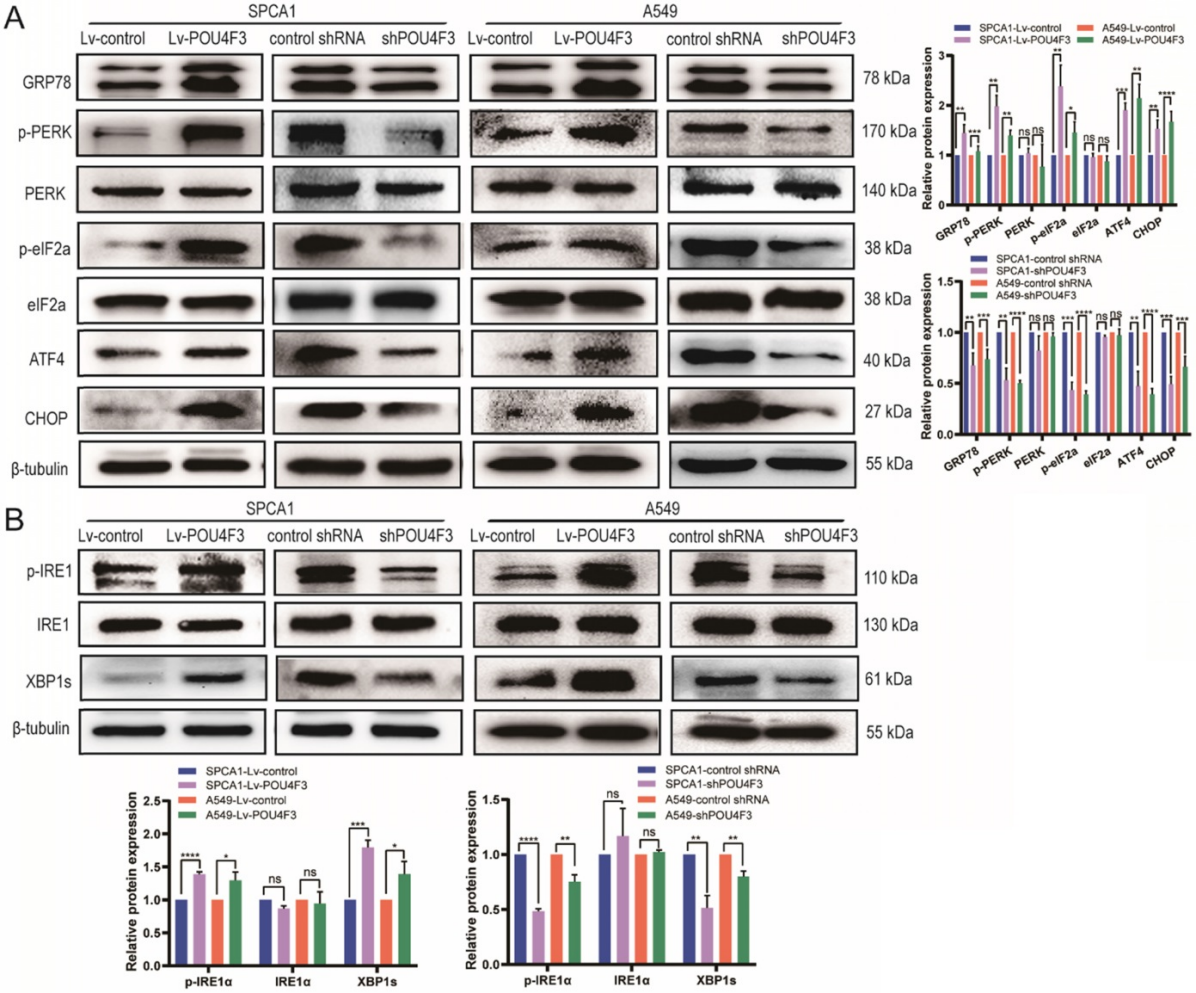
POU4F3 activated the ERS pathways in LUAD cells according to WB. A. The protein levels of PERK, p-PERK, eIF2α, p-eIF2α, ATF4, and CHOP in SPCA1 and A549 cells with or without POU4F3 overexpression, and with or without POU4F3 knockdown, were detected by WB. B. The protein levels of IRElα, p-IRElα, and XBP-1s in SPCA1 and A549 cells with or without POU4F3 overexpression, and with or without POU4F3 knockdown, were detected by WB.' ****', '***', and '**' represent *P* < 0.0001, 0.001, and 0.01 respectively.

Studies have shown that tunicamycin-induced apoptosis is associated with endoplasmic reticulum stress (ERS) 15, 16. We attempted to investigate whether POU4F3 affects LUAD cell apoptosis through ERS pathways. LUAD cells with stable POU4F3 overexpression or knockdown were given ERS inducer tunicamycin for 48 h. WB detected protein expression of ERS pathway molecules. POU4F3 overexpression elevated the expression of ERS key molecules GRP78, p-PERK, and p-IRE1α. On the first pathway, p-PERK increased downstream p-eIF2α, ATF4, and CHOP, indicating that overexpressed POU4F3 can activate the PERK/eIF2α/ATF4/CHOP pathway (*P* < 0.0001, Fig. 4A). On another pathway, POU4F3 overexpression increased p-IRE1α and downstream XBP-1s, suggesting that overexpressed POU4F3 can enhance the IRElα/XBP-1s pathway (*P* < 0.001, Fig. 4B). POU4F3 knockdown showed the opposite patterns in the changes of ERS molecules mentioned above. WB indicates that POU4F3 inhibits LUAD by activating the PERK/eIF2α/ATF4/CHOP and IRElα/XBP-1s pathways of ERS.

### PERK inhibitor but not IRE1 inhibitor can reverse the effect of POU4F3 overexpression on apoptosis

**Figure 5:**
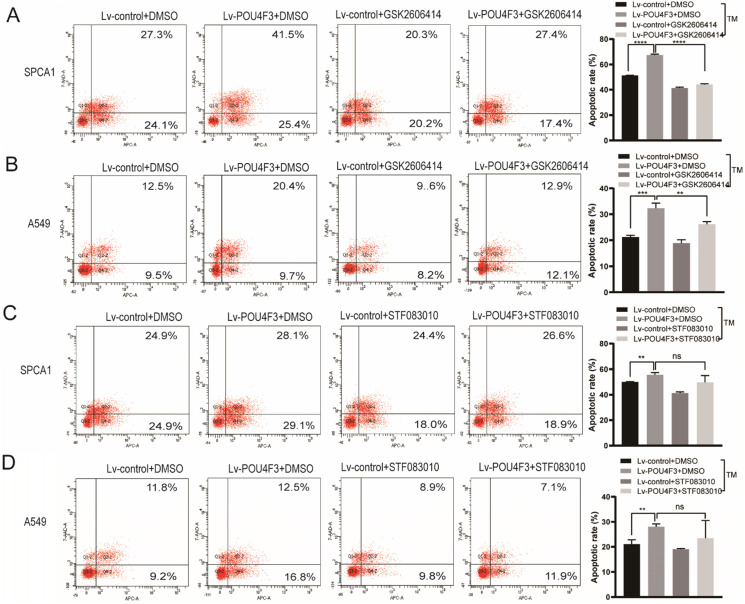
PERK inhibitor but not IRE1 inhibitor can reverse the effect of POU4F3 overexpression on apoptosis. Flow cytometry was used to detect the apoptotic rate in SPCA1 (A) and A549 (B) with or without the PERK inhibitor (GSK2606414). Flow cytometry was used to detect the apoptotic rate in SPCA1 (C) and A549 (D) with or without the IRE1 inhibitor (STF083010). ' ****', '***', and '**' represent *P* < 0.0001, 0.001, and 0.01 respectively.

To further confirm the role of PERK/IRE1 pathways in POU4F3 overexpression-induced promotion of apoptosis, the PERK inhibitor (GSK2606414) and the IRE1 inhibitor (STF083010) were utilized to attenuate the effect of POU4F3 overexpression on apoptosis. The apoptotic rate in the Lv-POU4F3+DMSO group was higher than in the Lv-control+DMSO group, both in regards to SPCA1 (*P* < 0.0001, Fig. 5A) and A549 cells (*P* < 0.001, Fig. 5B). Furthermore, the apoptotic rate in the Lv-POU4F3 group decreased after treatment by GSK2606414 (PERK inhibitor) in both SPCA1 (*P* < 0.0001, Fig. 5A) and A549 cells (*P* < 0.01, Fig. 5B). However, the apoptotic rate in the Lv-POU4F3 group was not changed after treatment with STF083010 (IRE1 inhibitor) in both SPCA1 (*P* > 0.05, Fig. 5C) and A549 cells (*P* > 0.05, Fig. 5D). Our results indicated that PERK inhibitor but not IRE1 inhibitor can reverse the effect of POU4F3 overexpression on apoptosis.

## Discussion

The POU transcription factor family is associated with a myriad of cancers, especially lung cancer. POU3F3 modulated cell proliferation, migration, and invasion in non-small cell lung cancer (NSCLC) 17. POU3F2 facilitated the invasiveness of small cell lung cancer (SCLC) 18. POU5F1 enhanced the migration and invasion of tumor stem cell-like cells in LUAD 19, and it predicted the poor prognosis of LUAD as a biomarker 20. The above members of the POU family appear to be more likely to affect lung cancer as oncogenes. However, the relationship between POU4F3 and lung cancer has not yet been elucidated.

In this study, IHC showed that POU4F3 was weaker in LUAD tissues than in normal tissues, which may suggest that POU4F3 was related to LUAD (Table 1). Consistently, POU4F3 is generally expressed in Merkel cells, but it was absent in Merkel cell carcinoma (MCC) 9. Further evaluation for POU4F3 and clinicopathological features of LUAD patients revealed that lower POU4F3 expression may be correlated with the higher TNM stage (Fig. 1B, D; Table 2). However, TNM stage IV did not follow this rule, possibly because there were only two cases in stage IV (Fig. 1B; Table 2). It is significant that LUAD patients with higher POU4F3 expression tended to have more prolonged overall survival (Fig. 1F). Also notable, the POU4F3 levels in LUAD patients are higher than those in normal controls according to the TCGA cohort (Supplementary 1A), which is contrary to the findings in our cohort study (Fig. 1D-F). The explanation is that our results were derived from protein levels in LUAD patients, whereas the TCGA database analysis was based on mRNA levels. Furthermore, the expression levels of POU4F3 were analyzed in various tumors in the TCGA database, such as hepatocellular carcinoma, adenocarcinoma of the colon, breast carcinoma, etc. (Supplementary 1A). We found that the expression of POU4F3 in hepatocellular carcinoma tissue was higher than that in adjacent liver tissues (*P* < 0.05), whereas there was no statistical difference in other types of cancers. Including LUAD, there was no statistical difference in the above cancers in terms of overall survival (Supplementary 1B).

Next, we constructed SPCA1 and A549 cells with stable POU4F3 overexpression or inhibition (Fig. 2A, B) to investigate the effect of POU4F3 on LUAD cells *in vitro* and* in vivo*. We discovered that the overexpressed POU4F3 attenuated the proliferation *in vitro* (Fig. 2C-F) and *in vivo* (Fig. 2G-H). We also explored the effect of POU4F3 on the cell cycle using the propidium iodide (PI) staining combined with flow cytometry (PI-FCM), but the results were not statistically significant (Supplementary 2). Additionally, POU4F3 promoted apoptosis (Fig. 3A-F) of LUAD cells, whereas POU4F3 knockdown yielded the reverse phenotype. Moreover, the apoptotic inhibitor Z-VAD rescued the apoptotic rate induced by POU4F3 overexpression in both LUAD cell groups (*P* < 0.01, Fig. 3G, 3H). Our experimental study suggested that POU4F3 may act as a suppressor in LUAD.

**Figure 6:**
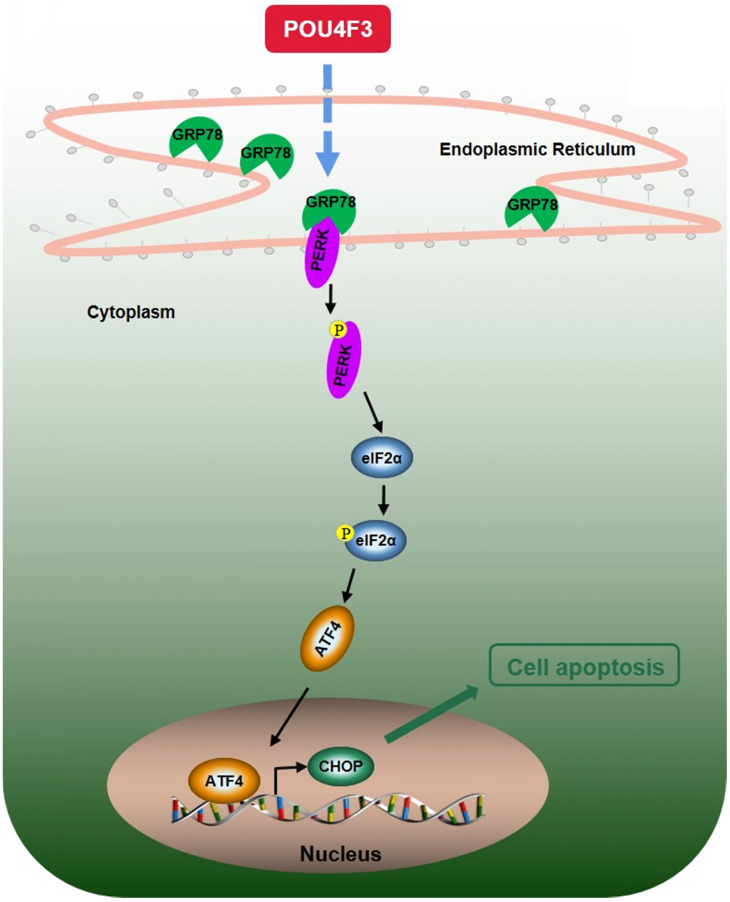
A simple diagram shows that POU4F3 suppresses LUAD via activating PERK/eIF2α/ATF4/CHOP pathway.

We further explored the possible mechanisms through which POU4F3 promoted the apoptosis of LUAD cells. Endoplasmic reticulum stress contains well-known signaling pathways associated with tumor proliferation 21 and apoptosis 22. Under severe or prolonged ER stress, apoptosis will be triggered and mainly mediated by the PERK and IRE1 pathways 23. GRP78, as a transmembrane molecular partner in the endoplasmic reticulum stress pathway, can specifically bind to transmembrane ER-stress sensors PERK and IRE1α in a non-stress state. When ERS occurs, GRP78 dissociates from the PERK and IRE1α dimerization and initiates autophosphorylation of PERK 24 and IRE1α 25. Our WB results showed that overexpressed POU4F3 in LUAD cells enhanced the key molecular GRP78 of the ERS pathways. Furthermore, POU4F3 intensified two ERS sub-pathways: PERK/eIF2α/ATF4/CHOP (Fig. 4A) and IRElα/XBP-1s (Fig. 4B). Using the PERK inhibitor (GSK2606414) and the IRE1 inhibitor (STF083010), we confirmed the role of PERK/eIF2α/ATF4/CHOP but not IRElα/XBP-1s pathway in POU4F3 overexpression-induced promotion of apoptosis (Fig. 5). Collectively, POU4F3 may increase apoptosis and consequently inhibit the proliferation of LUAD cells via the PERK/eIF2α/ATF4/CHOP pathway (Fig. 6). However, the mechanism by which POU4F3 activated the ERS signaling pathways needs to be further studied.

For the first time, our study demonstrated that POU4F3 is a novel tumor suppressor gene in LUAD. POU4F3 may become a prognostic target in LUAD. Increasing the expression of POU4F3 may be a potential therapeutic strategy for LUAD. Lastly, further translational research on the clinical application of POU4F3 is necessary.

## Supplementary Material

Supplementary figures.

## Figures and Tables

**Table 1:** Comparison of POU4F3 expression between LUAD and Normal^a^ groups.

	Number	POU4F3 expression		Positive rate (%)	*χ^2^*	*P* ^b^
		Positive	Negative			
Normal^b^	88	81	7	92.05	77.82	<0.0001
Luad	92	24	68	26.09		

a. Normal, normal lung tissue; LUAD, lung adenocarcinoma tissue. b. Chi-square was used to analyze the positive rate of POU4F3 expression. *P* < 0.05 was considered statistically significant.

**Table 2:** Basic Characteristics of Patients.

	N (%)	POU4F3 (n)		High expression rate (%)	*χ^2^*	*P* ^a^
		High	Low			
Age (years)					0.39	0.5315
<63	44(47.83)	24	20	54.55		
≥63	48(52.17)	22	26	45.83		
Gender					0.18	0.6751
Male	51(55.43)	24	27	47.06		
Female	41(44.57)	22	19	53.66		
Size (cm)					0.02	0.9021
>3.5	36(39.13)	18	18	50.00		
≤3.5	43(46.74)	22	21	51.16		
Location					0.01	0.8148
Upper lobe	47(61.84)	23	24	48.94		
Lower lobe	29(38.16)	13	16	44.83		
TNM Stage^b^					6.71	0.0348*
Ⅰ	25(37.31)	17	8	68.00		
Ⅱ-Ⅲ	40(59.70)	16	24	40.00		
Ⅳ	2(2.99)	2	0	100.00		
Differentiation grade^c^					0.19	0.6595
1-2	61(66.30)	32	29	52.46		
3	31(33.70)	14	17	45.16		
LNM^d^					0.97	0.2361
Positive	36(52.94)	16	20	44.44		
Negative	32(47.06)	19	13	59.38		
Prognosis					8.62	0.0031*
Survival	41(44.57)	28	13	68.29		
Death	51(55.43)	18	33	35.29		

a. Chi-square compared high expression rates of POU4F3. b. TNM stage was defined according to the seventh edition of AJCC clinical staging. c. The pathological differentiation grade was defined as follows: High-differentiated carcinoma (grade 1); Medium-differentiated carcinoma (grade 2); Low-differentiated carcinoma (grade 3). d. LNM: lymph node metastasis. *P < 0.05
