# Supplementary figures and images for: POU4F3 Acts as a Tumor Suppressor in Lung Adenocarcinoma via the Endoplasmic Reticulum Stress Signaling Pathway
Source: J Cancer. 2022 Jan 1;13(2):554–64. doi: 10.7150/jca.61660 (PMC8771506; doi:10.7150/jca.61660)

## Supplementary 1

A

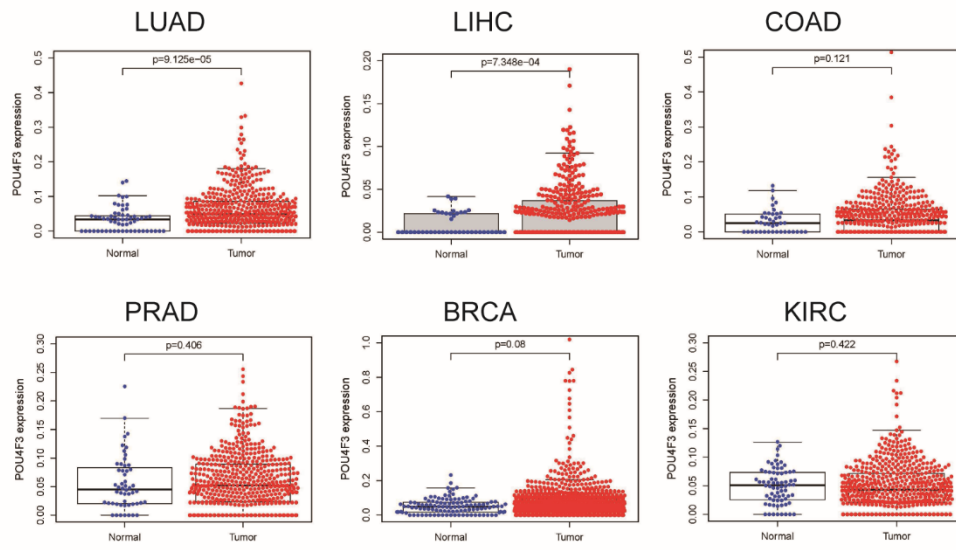

B

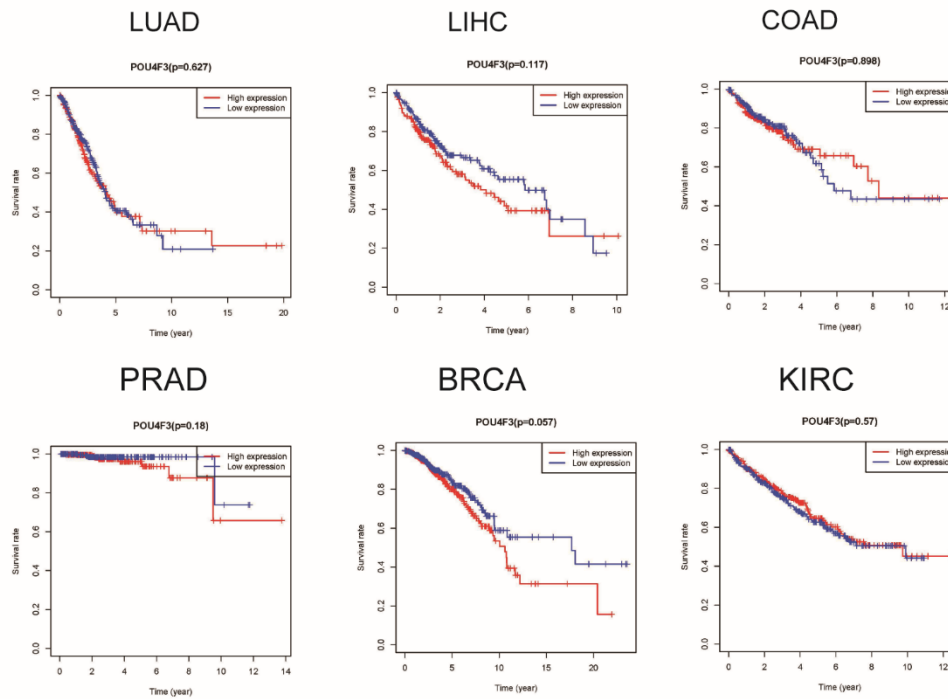

## Supplementary 2

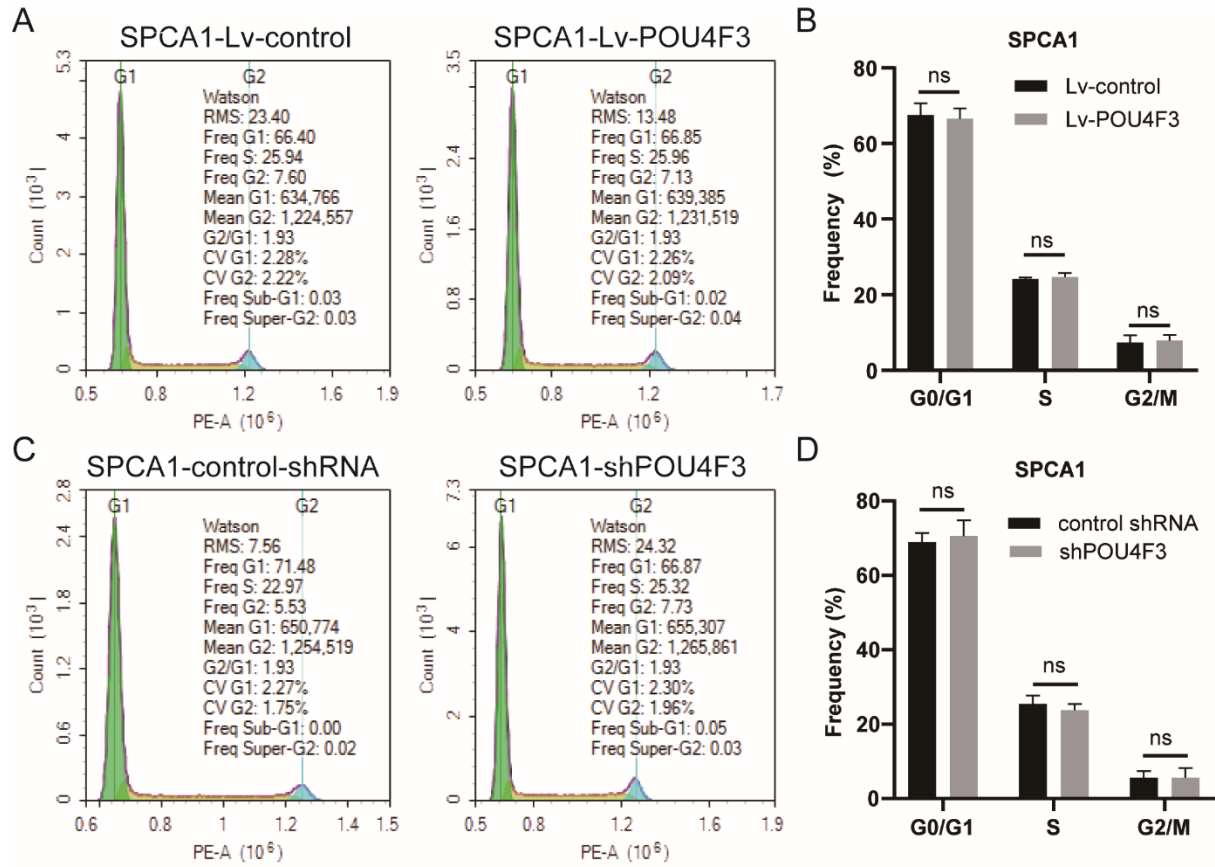

Supplement: Supplementary file 1 — Supplementary figures. [file jcav13p0554s1.pdf]
